# Autosomal Dominant Leukodystrophy: A Disease of the Nuclear Lamina

**DOI:** 10.3389/fcell.2019.00041

**Published:** 2019-03-20

**Authors:** Quasar S. Padiath

**Affiliations:** Department of Human Genetics, Graduate School of Public Health, University of Pittsburgh, Pittsburgh, PA, United States

**Keywords:** nuclear lamina, lamin B1, leukodystrophy, myelin, chromatin, lipid synthesis, nuclear structure

## Abstract

The nuclear lamina is a fibrous meshwork of proteins found adjacent to the inner nuclear membrane that plays a critical role in the maintenance of nuclear architecture. Made up of A and B type lamins, the nuclear lamina has recently been shown to contribute to numerous cellular functions such as chromatin organization, DNA replication, cellular proliferation, senescence, and aging. While at least a dozen disorders are associated with *LMNA*, the focus of this review is Autosomal Dominant Leukodystrophy (ADLD), the only disease associated with the lamin B1 gene (*LMNB1*). ADLD is a fatal, adult onset CNS demyelinating disorder that is caused by either genomic duplications involving *LMNB1* or deletions upstream of the gene. Both mutation types result in increased *LMNB1* gene expression. How the increased levels of this widely expressed nuclear structural component results a phenotype as specific as demyelination is a great mystery. This review summarizes what is currently known about the disease and describes recent work using animal and cell culture models that have provided critical insights into ADLD pathological mechanisms. The delineation of these pathways provides a fascinating glimpse into entirely novel roles for the nuclear lamina and will be critical for the identification of therapies for this fatal disease.

## The Nuclear Lamina and Disease

The nuclear lamina is a fibrous meshwork of intermediate filament proteins that is found adjacent to the inner nuclear envelope (Tatli and Medalia, 2018). Originally identified as a structural component critical for the maintaining the architecture of the nucleus, the nuclear lamina is now known to be involved in a diverse array of cellular and organismal processes. These include roles in cellular proliferation, senescence, aging, DNA replication and the 3D positioning of chromatin within the nucleus (Gruenbaum and Foisner, 2015; van Steensel and Belmont, 2017). Vertebrates possess two classes of Lamins, the A and B types. The A type lamins, lamin A and C are splice isoforms encoded for by a single gene *LMNA*. The B type lamins, Lamin B1 and B2 are encoded by separate genes *LMNB1* and *LMNB2* (Gruenbaum and Foisner, 2015).

Mutations in the *LMNA* gene are associated with at least 12 distinct disorders termed as laminopathies (Dobrzynska et al., 2016). Diseases involving the B type lamins are less common. Heterozygous mutations in lamin B2 are associated with an increased susceptibility to an acquired partial lipodystrophy (APL) also known as Barraquer–Simons syndrome, while homozygous mutations were identified in a single consanguineous family with progressive myoclonic epilepsy (Hegele et al., 2006; Damiano et al., 2015). The only disease associated with lamin B1, the focus of this review, is the fatal neurological disorder Autosomal Dominant Leukodystrophy (ADLD) (Padiath et al., 2006).

## Autosomal Dominant Leukodystrophy

Leukodystrophies are inherited neurological diseases that involve the myelin tracts in the central nervous system (CNS), where myelin involvement is the primary feature and not secondary to any underlying neuronal pathology (van der Knaap and Bugiani, 2017). While the exact prevalence of ADLD is unknown, it is likely to be an ultra-rare disease (Kohler et al., 2018). However, affected individuals have been described from around the world, suggesting that the disease is not specific to any specific ethnic group or geographic region (Nahhas et al., 2016).

**FIGURE 1:**
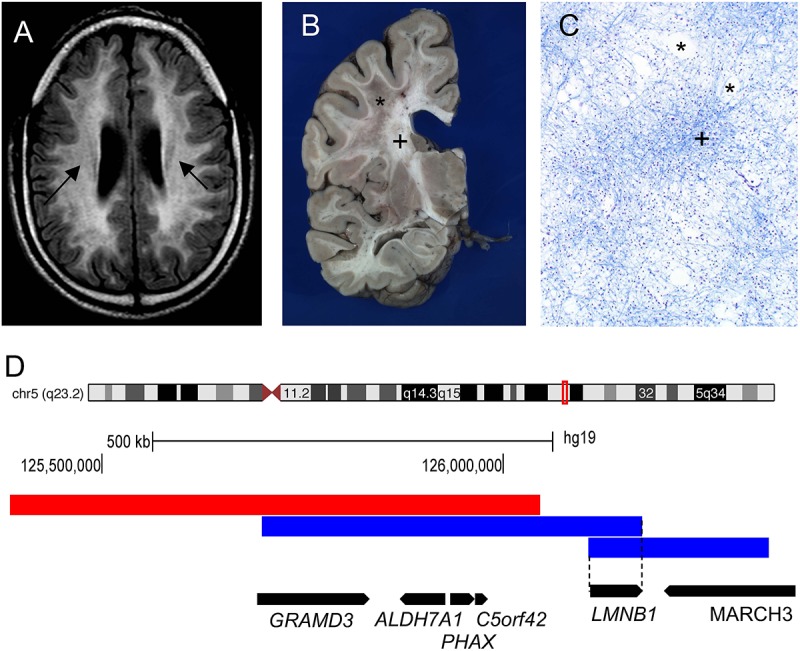
Genomic rearrangements involving the *LMNB1* gene cause the demyelinating disorder Autosomal Dominant Leukodystrophy (ADLD). **(A)** ADLD patient MRI (Fluid-attenuated inversion recovery sequence) reveals white matter hyperintensities indicating myelin pathology (marked by arrows). **(B)** ADLD patient brain showing patchy areas of myelin loss marked by asterisk (^∗^). Plus sign (+) indicates normal myelin. **(C)** Histopathological analysis of ADLD brain section using Luxol Fast Blue, a myelin stain, exhibits areas of pale staining and vacuolar demyelination, marked by asterisks (^∗^). Plus sign (+) indicates normal myelin staining. **(D)** Genomic region on chromosome 5q23.2 that contains the *LMNB1* gene. Blue bars indicate genomic duplications from two individual patients that have centromeric and telomeric junctions closest to the *LMNB1* gene that allow the identification of the minimal critical region required for disease causation (dashed line). Red bar indicates the deletion upstream of the *LMNB1* gene responsible for a variant ADLD phenotype reported in a single family. Data for **(D)** is modified from Giorgio et al. (2015). Black bars indicate the location of genes encompassed by the genomic rearrangements and the direction of transcription.

The age of onset in ADLD ranges from the fourth to fifth decade of life with MRI findings often preceding the onset of symptoms by many years. In the majority of the cases, signs of autonomic dysfunction such as bladder or bowel dysfunction, orthostatic hypotension, temperature dysregulation, and anhidrosis are the presenting features (Padiath and Fu, 2010; Finnsson et al., 2015; Nahhas et al., 2016). In a variant of ADLD (described below), autonomic dysfunction is not the first symptom and might be absent through the entire course of the disease (Brussino et al., 2009). Gait abnormalities, muscle weakness and spasticity usually follow with late stage dementia and cognitive impairment in some patients. ADLD is a fatal disease with patients surviving for one to two decades after the onset of symptoms (Finnsson et al., 2015). Magnetic resonance imagining (MRI) is the tool of choice for the diagnosis of ADLD with the presence of symmetrical cerebral white matter hyperintensities extending from the motor cortex to the medulla oblongata and the involvement of the upper and middle cerebellar peduncles being characteristic of the disease (Figure 1A; Finnsson et al., 2015). In the variant form of ADLD, a greater involvement of the frontal white matter with reduced cerebellar involvement was observed on the MRI (Brussino et al., 2009; Giorgio et al., 2015). Gross pathological analysis of ADLD brain specimens revealed patchy loss of white matter (Figure 1B) while histopathology showed a vacuolar demyelination phenotype (Figure 1C; Coffeen et al., 2000; Melberg et al., 2006; Alturkustani et al., 2013).

## Lamin B1 Mutations in Adld: Two Alternative Paths Leading to a Common End

Duplications involving the lamin B1 gene were identified to be the cause of ADLD (Padiath et al., 2006). This was the first and, till date, only disease phenotype associated with the *LMNB1* (Padiath and Fu, 2010).

A subsequent analysis of 16 unrelated ADLD families from various parts of the world revealed duplication events of different sizes, all of which encompassed the *LMNB1* gene, suggesting that duplications were caused by independent non-recurrent events (Figure 1D; Giorgio et al., 2013). In all but one case, the duplication events resulted in a head to tail tandem arrangement of the duplicated segments. In one patient, the duplicated region was inverted and inserted upstream of the original genomic segment (Giorgio et al., 2013). The presence of microhomology of between 1 and 10 bp at the duplication junctions and the non-recurrent nature of the duplication events strongly suggested that these genomic rearrangements were mediated either by Non-Allelic Homologous Recombination (NAHR) or replication mediated errors such as Microhomology Mediated Break Induced Repair (MMBIR) (Hastings et al., 2009). Subsequent to the identification of *LMNB1* duplications as the genetic cause underlying ADLD, a 600 Kb deletion upstream of the *LMNB1* gene was reported in a single large Italian pedigree with an adult onset demyelinating phenotype similar to ADLD (Figure 1D). In these patients, autonomic dysfunction was either absent or present only at the later stages of the disease (Giorgio et al., 2015). The demyelination phenotype in ADLD can thus be caused by two distinct mechanisms: tandem duplications involving the *LMNB1* gene or deletions upstream of the *LMNB1* gene.

In both duplication and upstream deletion cases, the common pathological mechanism is thought to be increased *LMNB1* expression. The initial description of the ADLD mutation identified increased expression of both *LMNB1* mRNA and protein from ADLD brain tissue (Padiath et al., 2006). Subsequent reports demonstrated increased *LMNB1* expression in fibroblasts and blood derived from ADLD patients when compared to age matched controls (Giorgio et al., 2013). *LMNB1* overexpression was also demonstrated in the fibroblasts from patients with the variant form of ADLD, caused by the upstream deletions, although this did not reach the same magnitude as observed in the duplication patients (Giorgio et al., 2015).

The lack of significant autonomic dysfunction in the upstream deletion cases is striking and suggests that there might be subtle differences in overexpression patterns between the deletion and duplication. This hypothesis was supported by findings of a greater magnitude of overexpression in the frontal lobe in a sample of brain tissue from a patient with the upstream deletion (Figure 1D; Brussino et al., 2009; Giorgio et al., 2015). To explain this finding, it was suggested that the deletion upstream of the *LMNB1* genes resulted in an enhancer adoption mechanism whereby a forebrain enhancer was brought in close proximity of the *LMNB1* gene, resulting in overexpression that was more pronounced in the frontal white matter (Giorgio et al., 2015).

## Mouse Models of Adld

**FIGURE 2:**
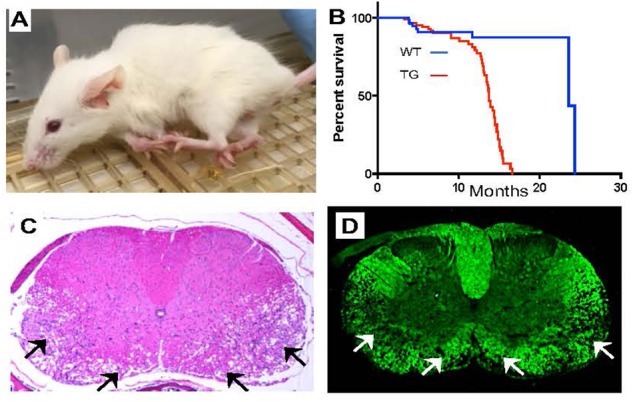
Mouse models overexpressing *LMNB1* recapitulate ADLD phenotypes. **(A)** Transgenic mice where LMNB1 is targeted to oligodendrocytes show progressive age dependent muscle wasting, kyphosis, limb paralysis and **(B)** significantly reduced lifespan. Histopathological analysis of spinal cord sections from transgenic mice reveal severe vacuolar degeneration of myelinated regions (arrows). **(C)** Hematoxylin and Eosin staining. **(D)** Fluoromyelin (a myelin specific stain) staining [Reproduced with permission from Rolyan et al. (2015)].

Mouse models engineered to overexpress *LMNB1* have proved invaluable in advancing our understanding of the disease (Padiath, 2016). Transgenic mice, where lamin B1 cDNA overexpression was specifically targeted to oligodendrocytes (*Plp-LMNB1*), the cell types that produce CNS myelin, were normal at birth but exhibited profound age dependent motor dysfunction including ataxia and forelimb paralysis by about 8 months of age, reminiscent of the late age dependent onset of the human disease (Figure 2A). These mice showed a significant increase in mortality and most did not survive beyond 13–15 months of age (Heng et al., 2013; Rolyan et al., 2015; Figure 2B). Interestingly, overexpression of lamin B1 in astrocytes or neurons did not produce any observable phenotype, suggesting that oligodendrocytes are probably the dominant cell type in the disease (Heng et al., 2013). A mouse model where overexpression was driven by a mouse BAC transgene containing the *LMNB1* and all its upstream regulatory elements produced a much milder phenotype, with mice surviving beyond 2 years of age, although age dependent motor decline and cognitive impairments involving spatial memory deficit were also reported (Heng et al., 2013).

A detailed histopathological examination of the older *Plp-LMNB1* mice demonstrated significant age dependent vacuolar demyelination, similar to what was observed in the patients, most prominent in the spinal cord (Rolyan et al., 2015; Figure 2C,D). Electron microscopy analysis also revealed a significant disruption of the normal myelin architecture in older *Plp-LMNB1* mice, with an increase in degenerated and redundant myelin. Thinner myelin, indicative of remyelinated axons was also observed (Heng et al., 2013; Rolyan et al., 2015). An analysis of oligodendrocyte cell number did not reveal any reduction in these mice, nor was there an increase in markers of apoptosis, suggesting that the overexpression of lamin B1 is not detrimental to cell survival. Axonal damage and neuronal cell death were also observed in the *Plp-LMNB1* mice; however, these were thought to be secondary to the demyelination phenotype as they were observed only in later stages of the disease (Rolyan et al., 2015).

Rolyan et al. (2015), suggested that the primary involvement of the spinal cord in the *Plp*-*LMNB1* mice was more a function of the spatial expression patterns of the *Plp1* promoter, which drives expression highest in the spinal cord, rather than any mechanistic differences between the mouse model and the human disease (Fuss et al., 2000). Indeed, the vacuolar demyelination observed in the *Plp*-*LMNB1* mice was quite similar to that observed in humans. This dependence of the location of the pathology upon the levels of lamin B1 overexpression is consistent with the hypothesis put forward for the difference between the ADLD phenotypes produced by *LMNB1* duplications and upstream deletions (Giorgio et al., 2015). It is possible that different sizes or orientations of genomic rearrangements, be they deletions or duplications, might result in subtle differences in lamin B1 overexpression patterns leading to phenotypic variation. An accurate characterization of the genomic rearrangements associated with ADLD is thus critical.

Although oligodendrocytes clearly play a critical role in the pathology of ADLD, the contribution of other CNS cell types to the disease phenotype cannot be entirely discounted. Autonomic dysfunction, which is a major clinical feature in ADLD was not observed in the *Plp*-*LMNB1* mice suggesting that other cell types may be responsible for these symptoms (Lo Martire et al., 2018). Support for the involvement of non-oligodendrocyte CNS cells comes from both *in vivo* and *in vitro* data. Histopathological analysis of ADLD brain tissue revealed astrocytes with significantly shortened and beaded processes, suggesting that a primary astrocyte pathology might also be present in the disease (Coffeen et al., 2000; Melberg et al., 2006). *In vitro* studies where lamin B1 was transiently overexpressed in cortical neurons resulted in significant reduction in axonal length (Giacomini et al., 2016). Neuronal or glial overexpression of lamin B1 in *Drosophila* resulted in significant lethality, while overexpression in the fly eye led to a severe degenerative phenotype (Padiath et al., 2006). These reports suggest that astrocytes and neurons might also be susceptible to the deleterious effects of *LMNB1* overexpression and further studies are thus required to determine the role of non-oligodendrocyte cells in ADLD.

## Lamin B1 Mediated Transcriptional Dysregulation and Disruption of Lipid Synthesis in Adld Mouse Models

The lack of oligodendrocyte cell death in both the human disease and mouse models clearly indicate that the overexpression of lamin B1 is not deleterious to cell survival. The fact that both humans and mice with *LMNB1* overexpression produce normal myelin, which is only lost later in adulthood, strongly indicates that pathways responsible for myelin maintenance in the oligodendrocytes are compromised.

One of the pathways implicated was a reduced expression of the *Plp1* gene which encodes a transmembrane protein that makes up ∼50% of CNS myelin. Heng et al. (2013) demonstrated that *Plp1* expression was reduced in the BAC transgenic and *Plp*-*LMNB1* transgenic mice. They also demonstrated reduced of binding YY1, a known transcriptional activator of *Plp1* as a potential mechanism to explain reduced expression of this gene in *LMNB1* BAC mice. However, given that the *Plp1* null mice do not exhibit demyelination but only axonal degenerative phenotypes (Griffiths et al., 1998), it was suggested that the reduction in *Plp1* expression was insufficient to explain the degenerative phenotype in the ADLD mice, but was more likely to serve as an initial trigger for the disease (Heng et al., 2013).

An independent analysis of the *Plp*-*LMNB1* mice revealed significant age dependent reductions in the expression of multiple genes responsible for lipid synthesis in oligodendrocytes, some well before the disease onset, suggesting a primary causative role (Rolyan et al., 2015). This reduction in gene expression was thought to be driven by progressive increases in epigenetic histone modifications that favored transcriptional repression (H3K9me3 and H3k27me3), accompanied by reductions in histone modifications associated with transcriptionally active chromatin (Ach3 and Ach4). Interestingly, this study did not identify any reduction in the expression levels of genes encoding the major myelin proteins such as Proteolipid Protein 1 (PLP1) or Myelin Basic Protein (MBP) suggesting that the transcriptional repression was selective for genes in lipid synthesis pathways. Functional consequences of reduced gene expression were confirmed by a lipidomics analysis of the affected tissues which demonstrated significant reductions in multiple classes of myelin enriched lipids such as cholesterol and various phospholipid species. Given that lipids constitute 70% of myelin, Rolyan et al. (2015) concluded that the reduced lipid synthesis was a major driver of the demyelination phenotype. As oligodendrocytes are required to synthesize extremely large quantities of lipids in order to generate myelin, the authors also hypothesized that these cells might be uniquely susceptible to effects of lamin B1 mediated lipid dysregulation thereby accounting for the specificity of the demyelination phenotype.

## Cell Culture Models of Adld Implicate Diverse Molecular Pathways

Further insights into possible disease mechanisms have come from the study of cell culture models that overexpress lamin B1 (Table 1). Transient overexpression of *LMNB1* in immortalized cells resulted in abnormal nuclear morphology (Padiath et al., 2006) and increased folding of the nuclear envelope together with alterations in the localization of the nuclear membrane protein LAP2, heterochromatin protein 1β (HP1β) and methylated histone 3 (K3H9), while similar experiments in primary oligodendrocytes resulted in a reduced expression and redistribution of major myelin proteins (Lin and Fu, 2009). The effect of lamin B1 overexpression on oligodendrocytes could be mitigated by the concomitant expression of *mir-23a*, a LMNB1 targeting micro RNA that reduced LMNB1 levels (Lin and Fu, 2009).

**Table 1:** Pathways implicated in the pathogenesis of ADLD.

Pathway	Type of model used	Reference
Transcriptional dysregulation	Cell culture, Mouse model	Lin and Fu, 2009; Heng et al., 2013; Rolyan et al., 2015
Alteration of histone modifications	Mouse model	Rolyan et al., 2015
Reduced lipid synthesis	Mouse model	Rolyan et al., 2015
Nuclear structural abnormalities and nuclear signaling	Cell culture	Padiath et al., 2006; Lin and Fu, 2009; Ferrera et al., 2014
Alterations in RNA splicing	Cell culture	Bartoletti-Stella et al., 2015
Transcription factor sequestration	Cell culture	Columbaro et al., 2013
Impaired response to Reactive Oxygen Species (ROS)	Cell culture	Barascu et al., 2012; Columbaro et al., 2013


Skin fibroblasts from ADLD patients, where *LMNB1* was found to be overexpressed by ∼1.5–2 fold, displayed nuclear abnormalities that were much more subtle and did not reveal a mislocalization of LAP2β or nucleoporins (Ferrera et al., 2014). These contradictory results were attributed to the fact that transient transfection might result in the expression of the *LMNB1* protein that were much higher than physiological levels. This report also revealed that ADLD fibroblasts exhibited increased nuclear rigidity and reduced nuclear ion channel open probability on voltage-step application. The authors postulated that because the nucleus acts as a mechanosensor, modulating gene expression in response to internal and external forces, the altered mechanical properties of *LMNB1* overexpressing cells might impact downstream signaling, thereby contributing to the disease phenotype (Ferrera et al., 2014).

In an independent study, ADLD fibroblasts were found to sequester OCT1, a transcription factor that regulates genes that are essential for the cellular response to oxidative stress. Lamin B1 was previously shown to bind OCT1 and in ADLD cells OCT1 staining was markedly increased at the nuclear rim. In response to oxidative stress (H_2_O_2_ treatment) OCT1 relocalization to the nucleoplasm was significantly reduced in ADLD cells (Malhas et al., 2009; Columbaro et al., 2013). Lamin B1 knockout cells were previously shown to accumulate higher levels of reactive oxygen species (ROS) (Malhas et al., 2009) and it would be interesting to determine whether the overexpression of lamin B1 in disease relevant cells such as oligodendrocytes renderers them more susceptible to the toxic effects of ROS. The link between ROS and *LMNB1* is an important one as ROS levels have been shown to increase in the brain with age (Stefanatos and Sanz, 2018) and a recent report has suggested that elevated ROS can lead to an accumulation of *LMNB1* (Barascu et al., 2012). One can imagine a scenario whereby lamin B1 levels, which are already higher in ADLD patients is further increased by the age dependent ROS accumulation to a critical threshold beyond which cellular dysfunction ensues, thus accounting for the age dependence of the disease.

Yet another pathway that has been implicated in ADLD is one involving RNA splicing (Bartoletti-Stella et al., 2015). Microarray analysis of blood and fibroblast RNA from ADLD patients identified the expression of the ribonucleoprotein PTB-binding protein 2 (*RAVER2*) to be increased in both tissue types relative to controls. *Raver2* was conversely reduced in lamin B1 null embryos. Polypyrimidine tract binding protein (PTB) is splicing factor involved in all steps of mRNA metabolism and acts as repressor of alternatively spliced exons and the relative splice isoforms levels of eight PTB target genes were found to be altered in the ADLD fibroblasts (Kleinhenz et al., 2005; Bartoletti-Stella et al., 2015). The authors also identified altered splicing of the transcript from gene encoding the major myelin protein, *PLP1*. There was a reduction in the adult isoform of *PLP1* in comparison to *DM20*, the embryonic isoform, in ADLD fibroblasts when compared to controls. The opposite result was observed in brain tissue from lamin B1 null embryos, where there was an increase of *Plp1/Dm20* ratio when compared to control animals. The authors proposed that ADLD could be a spliceopathy caused by a LMNB1 dependent increase in *RAVER2*, resulting in an increase in the embryonic form of *PLP1* during adulthood, contributing to the demyelination phenotype (Bartoletti-Stella et al., 2015).

## Summary

Autosomal Dominant Leukodystrophy provides a fascinating glimpse into the diverse and often unexpected functions of the nuclear lamina and the importance of nuclear architecture and chromatin regulation in health and disease. The development of multiple mouse and cell culture models have contributed significant insights into the pathogenesis of ADLD and provided tantalizing clues as to how a widely expressed protein such as Lamin B1 can be involved in a specific pathology such as demyelination (Table 1). However, significant work needs to be carried out to delineate the pathways linking *LMNB1* and myelin regulation and determine the relative contribution of each of these to the disease phenotype. This is not only important for understanding the basic biology of the ADLD but will also be critical in identifying therapeutic interventions for this fatal disorder.

## Author Contributions

The author confirms being the sole contributor of this work and has approved it for publication.

## Conflict of Interest Statement

The author declares that the research was conducted in the absence of any commercial or financial relationships that could be construed as a potential conflict of interest.
